# Naturerfahrung und seelische Gesundheit bei Kindern – theoretische Ansätze und ausgewählte empirische Befunde

**DOI:** 10.1007/s00103-023-03729-w

**Published:** 2023-06-06

**Authors:** Ulrich Gebhard

**Affiliations:** Fakultät für Erziehungswissenschaft, AG 4, Universität Bielefeld, Postfach 10 01 31, 33501 Bielefeld, Deutschland

**Keywords:** Salutogene Wirkung von Naturerfahrungen, Stress, Kognitive Entwicklung, Selbstwert, Bewegung, ADHS, Naturpädagogik, Salutogenic effect of nature experiences, Stress, Cognitive development, Self-esteem, Exercise, ADHD, Nature education

## Abstract

Die vielfach belegten günstigen Effekte von Naturerfahrungen bei Kindern machen die Annahme plausibel, dass sich eine naturnahe Umgebung auch positiv auf die Gesundheit im Kindesalter auswirkt, also auch ein Beitrag zur Gesundheitserhaltung und -prävention sein kann. Die Befunde zu gesundheitsfördernden Wirkungen von Natur sind bemerkenswert und werden hier mit dem Fokus auf seelische Gesundheit akzentuiert und theoretisch fundiert.

Grundlage ist ein sogenanntes dreidimensionales Persönlichkeitsmodell, demzufolge die seelische Entwicklung nicht nur eine Funktion der Beziehung des Subjekts zu anderen Menschen, sondern auch zur Welt der Dinge, also auch der Natur ist. Es werden zudem 3 Erklärungsansätze für die gesundheitlichen Wirkungen von Naturerfahrungen skizziert: 1. die anthropologisch fundierte „Stress Recovery Theory“, 2. die „Attention Restoration Theory“ und 3. die Annahme, dass Natur als ein Symbolvorrat für Selbst- und Weltdeutungen die Sinnkonstituierung der Subjekte begleiten kann („therapeutische Landschaften“).

Es wird v. a. auf die gesundheitlichen Wirkungen von erreichbaren naturnahen Freiräumen eingegangen, wobei der Forschungsstand für Erwachsene weitaus reichhaltiger ist als für Kinder. Im Hinblick auf die seelische Gesundheit bzw. auf deren Einflussgrößen werden die folgenden Dimensionen mit empirischen Ergebnissen ausgeführt: Stressreduktion, antidepressive und stimmungsaufhellende Wirkung, prosoziales Verhalten, Aufmerksamkeit und ADHS (Aufmerksamkeitsdefizit-Hyperaktivitätsstörung), kognitive Entwicklung, Selbstwert und Selbstregulation, Naturerfahrung und Bewegung. Aus salutogenetischer Sicht wirkt Natur nicht deterministisch auf die Gesundheit, sondern gewissermaßen beiläufig, wenn naturnahe Freiräume erreichbar sind und genutzt werden. Diese Beiläufigkeit der Wirkung von Naturerfahrungen ist bei möglichen therapeutischen oder pädagogischen Interventionen zu bedenken.

## Abstract (en)

The widely documented beneficial effects of children’s experiences with nature make it plausible to assume that an environment close to nature also has a positive effect on health in childhood, that is, that it can also contribute to health maintenance and prevention. The findings on health-promoting effects of nature are remarkable and are accentuated and theoretically substantiated here with a focus on mental health.

The basis is a so-called three-dimensional personality model, according to which mental development is not only a function of the subject’s relationship to other people, but also to the world of things, including nature. In addition, three explanatory approaches for the health effects of nature experiences are outlined: (1) the anthropologically based “Stress Recovery Theory,” (2) the “Attention Restoration Theory,” and (3) the assumption that nature as a symbolic storehouse for self- and world interpretations can accompany the meaning constitution of the subjects (“Therapeutic Landscapes”).

The health effects of accessible open spaces close to nature are discussed, whereby the state of research for adults is much richer than for children. With regard to mental health or its influencing variables, the following dimensions are elaborated with empirical results: stress reduction, antidepressant and mood enhancing effects, prosocial behavior, attention and ADHD, cognitive development, self-esteem and self-regulation, nature experience, and exercise. From a salutogenetic perspective, nature does not have a deterministic effect on health, but rather, in a sense, an incidental effect when open spaces close to nature are accessible and used. This casualness of the effect of nature experiences has to be considered in possible therapeutic or educational interventions.

## Naturerfahrung und Gesundheit

Die vielfach belegten günstigen Effekte von Naturerfahrungen bei Kindern [1, 2] machen die Annahme plausibel, dass eine naturnahe Umgebung sich auch positiv auf die Gesundheit auswirkt. Bisweilen wird sogar von einem „Nature Deficit Syndrom“ gesprochen [3] und damit der Zusammenhang von Naturerfahrung und gesunder Entwicklung im Kindesalter besonders hervorgehoben.

Eine besondere Rolle spielt dabei die sog. Naturverbundenheit [4]. Diese ist ein wichtiges Element des Selbstkonzepts und korreliert mit der Fähigkeit zur Perspektivenübernahme und auch mit dem Umweltbewusstsein. So schreiben Ryan und Deci [5] der Naturverbundenheit eine energetisierende und vitalisierende Wirkung zu [6]. Wichtig ist dabei die Unterscheidung von Erlebnis und Erfahrung (Dewey): Erst ein Erlebnis, das uns (auf oft irritierende Weise) berührt, die Phantasie anregt und auch reflexiv gewendet wird, kann uns in unserer Persönlichkeit nachhaltig berühren [1].

Die zentrale Annahme dieses Aufsatzes ist, dass die Möglichkeit oder das Angebot von Naturerfahrungen auch ein Beitrag zur Gesundheitserhaltung für Kinder sein kann [7, 8]. Angesichts dieser Präventionsfunktion geht es auch um mögliche (pädagogische) Fördermaßnahmen, um Kindern einen Zugang zur Natur zu erschließen (s. Abschnitt „Beiläufige Wirkung von Naturerfahrungen und naturpädagogische Einflussmöglichkeiten“).

Auch in die Public-Health-Debatte haben naturnahe Grünräume v. a. im Hinblick auf die Stadtplanung [9] Einzug gehalten. Naturräume mit Wiesen, Feldern und Wäldern haben einen belebenden Effekt und bewirken eine Erholung von geistiger Müdigkeit und Stress. Dies gilt auch für Kinder [10].

Der Zusammenhang von Naturerfahrungen und Gesundheit wird häufig mit evolutionären Annahmen in Verbindung gebracht, wonach das Präferieren von naturnahen Umwelten und auch die gesundheitlichen Wirkungen mit biologisch fundierten Dispositionen zusammenhängen („Biophilie“ [11]). Nach der „psychoevolutionären“ Theorie [12] präferieren Menschen Naturumwelten, die in der Phylogenese des Menschen gleichsam überlebenswirksam gewesen sind. Solche Umwelten zeichnen sich durch eine Kombination von sicherheitsinduzierenden Merkmalen (Schutz, kleine Baumgruppen, Wasser, Gras) einerseits und Explorationsanreizen andererseits aus (Prospect-Refuge- bzw. Savannen-Theorie). Diese Kombination ist danach in natürlichen Umwelten eher gegeben als in bebauten. Die besagte Kombination führt durch eine Aktivierung des parasympathischen Nervensystems zu einer Stressreduktion (Stress Recovery Theory) und befördert positive Emotionen bzw. reduziert negative Emotionen [12].

Nach der Attention Restoration Theory [13] wirken sich Naturräume deshalb günstig auf die Gesundheit aus, weil sie eine Erholung verbrauchter Aufmerksamkeitskapazität bewirken [14]. Zudem ermöglichen Naturerfahrungen einen Abstand zum Alltagsleben und sie provozieren Aufmerksamkeit, die nicht anstrengt.

„Natürlich“ wirkt „Natur“ nicht automatisch entlang von biologisch-anthropologischen Konstanten. Die jeweiligen Bedeutungen von Natur und Landschaft werden nämlich vor dem Hintergrund kultureller Rahmungen auch subjektiv erzeugt (s. Abschnitt „Natur als salutogener Faktor“).

## Selbst und Welt – die psychodynamische Bedeutung von nichtmenschlicher Umwelt und Natur

Die Annahme einer Korrespondenz zwischen seelischer Gesundheit und Umwelt impliziert Annahmen über den Einfluss der nichtmenschlichen Umwelt auf die seelische Entwicklung. In den meisten Persönlichkeitsmodellen hängt jedoch die psychische Entwicklung v. a. von der Art und Qualität der menschlichen Umwelt ab. Wie wichtig z. B. feste Bezugspersonen für die Entwicklung in der (frühen) Kindheit sind, ist unbestritten. Die Erfahrungen, die Kinder in den ersten Lebensjahren mit vertrauten Bezugspersonen machen, bestimmen wesentlich die Persönlichkeit und auch, mit welcher Tönung und Qualität die Welt wahrgenommen wird. Macht das Kind die Erfahrung, dass es geliebt und gewollt ist und dass es gehalten wird, so sind das gute Bedingungen für ein von Vertrauen geprägtes Verhältnis zur Welt, zu anderen Menschen und auch zu sich selbst („Urvertrauen“). Diese Vertrautheit hat etwas zu tun mit dem Vertrautwerden und mit dem Vertrauen, das wir im Kontext unserer primären Beziehungen zu Menschen erfahren haben. Dieses gewissermaßen zweidimensionale Persönlichkeitsmodell interpretiert die psychische Genese als das Ergebnis der Beziehung zwischen dem Subjekt und anderen Menschen.

Hier nun geht es um die Bedeutung der Natur für die Konstituierung eines solchen Vertrauens. Es geht um den Gedanken, dass dieses sich auch als das Ergebnis einer gelungenen Beziehung zur Natur, überhaupt zur Welt der Dinge verstehen lässt, dass unser Leben also im Sinne des Wortes „bedingt“ ist [15]. Dinge sind nicht nur objektive Gegebenheiten, sondern in gewisser Weise auch Interaktionspartner; dadurch werden sie zu Elementen eines persönlich gedeuteten Lebens und erhalten damit eine emotionale Bedeutung. Diese Bedeutung haftet symbolisch den Dingen an, womit sie Ausdruck unserer Deutungsmuster gegenüber der Welt sind. Die Vertrautheit mit den Dingen, auch mit der Natur, konstituiert also ein basales Weltbild, das etwas mit unserem Lebensgefühl und auch mit seelischer Gesundheit zu tun hat. Dadurch kann bei der Erfahrung von Natur atmosphärisch viel mehr mitschwingen als die neutrale Registrierung von Objekten und so etwas wie besagtes Urvertrauen begründen. Analog zum Konzept der Bezugspersonen könnte man hier auch von „Bezugsdingen“ oder von „Bezugsorten“ sprechen [16].

Während es bezüglich der biologisch-ökologischen Verflochtenheit des Menschen mit der nichtmenschlichen Natur keine Zweifel mehr geben kann, suggeriert ein zweidimensionales Persönlichkeitsmodell, dass man sich die psychische Genese der menschlichen Persönlichkeit unabhängig von der nichtmenschlichen Umwelt vorstellen könne. Insofern ist das zweidimensionale Persönlichkeitsmodell durch die dritte Dimension zu erweitern, um die Beziehung des Menschen auch mit der nichtmenschlichen Umwelt und damit auch der Natur in den Blick zu nehmen.

Die Psychoanalyse ist ein klassisches Beispiel dafür, wie die Genese von Persönlichkeitsstrukturen (und -störungen) v. a. aus intra- und interpsychischen Prozessen abgeleitet wird. In der Objektbeziehungstheorie der Psychoanalyse sind die relevanten „Objekte“ immer Menschen. Der einzige konsistente psychoanalytische Ansatz, der die Bedeutung der nichtmenschlichen Umwelt für die Persönlichkeitsentwicklung reflektiert, ist die Arbeit von H. F. Searles [17]. Searles postuliert eine grundlegende „Verwandtschaft“ des Menschen mit der nichtmenschlichen Umwelt. Diese bestehe zwischen Mensch und nichtmenschlicher Umwelt bereits vor jeder konkreten psychischen Erfahrung oder Entwicklung. Diese Verwandtschaft konstituiert den Rahmen, innerhalb dessen psychische Entwicklung sich vollziehen kann.

Searles schlägt eine (neue) psychoanalytische Entwicklungslehre vor, die auf der Grundlage klassischer psychoanalytischer Theorieelemente (v. a. der Objektbeziehungs- und der Narzissmustheorie) reflektiert, welchen Einfluss die nichtmenschliche Umwelt auf die seelische Entwicklung hat. Schon Freud hat zum Verhältnis von Ich und Welt einen ähnlichen Gedanken formuliert: „Ursprünglich enthält das Ich alles, später scheidet es eine Außenwelt von sich ab. Unser heutiges Ichgefühl ist also nur ein eingeschrumpfter Rest eines weit umfassenderen, ja – eines allumfassenden Gefühls, welches einer innigeren Verbundenheit des Ichs mit der Umwelt entsprach“ [18, S. 424 f.]. Dieses Gefühl der allumfassenden Verbundenheit mit der Umwelt nennt Freud „ozeanisch“. Die psychische Leistung, zwischen sich selbst und der nichtmenschlichen Umwelt differenzieren zu können, ist Searles zufolge als ein entscheidender Entwicklungsschritt anzusehen, ähnlich wie die Lösung aus der symbiotischen Mutterbeziehung.

Das Neue an diesem Gedankengang ist grundlegend. Wenn es richtig ist, dass die Erfahrung, die das Kind mit den primären Objekten macht, wesentlich die spätere Persönlichkeit, das Lebensgefühl, das „Urvertrauen“ bestimmt, dann wird eben dieses Lebensgefühl auch von der Art und Qualität der nichtmenschlichen Umwelt geprägt sein. Dieses basale Gefühl konstituiert sich aus der Erfahrung der gelungenen und als befriedigend erlebten Beziehung zu den primären Objekten: Das sind Menschen, Gegenstände, Pflanzen, Tiere, Häuser, Landschaften usw.

## Naturerfahrung als Element des Wohlbefindens und der Gesundheit von Kindern

Die Erfahrung von äußerer Natur ist also auch bedeutsam für die Entwicklung der inneren Natur des Menschen. Insofern kann die Möglichkeit bzw. das Angebot von Naturerfahrungen auch ein Beitrag zur Gesundheitserhaltung sein.

Die günstigen Wirkungen von Naturerfahrungen für die Entwicklung von Kindern können als wichtiger Hintergrund betrachtet werden, durch den auch die Verbindung von Naturerfahrung und Gesundheit bzw. Wohlbefinden plausibel wird. Dieser Zusammenhang wird wesentlich in der frühen Kindheit angebahnt. Wer als Kind die Gelegenheit zu Naturerfahrungen hatte, kann eben dies als Ressource auch in späteren Lebensphasen nutzen [19]. In diesem Aufsatz wird v. a. auf die gesundheitliche Wirkung von erreichbaren naturnahen Freiflächen eingegangen. Die ebenfalls gut untersuchten Wirkungen des Kontakts zu Tieren sind nicht Gegenstand dieses Artikels. Und auch die Wirkungen von Gärten bzw. Pflanzen werden nicht behandelt.

Insgesamt sind die günstigen Wirkungen von Naturerfahrungen auf Gesundheit und Wohlbefinden („well being“) empirisch gut untersucht, wobei sich die meisten Studien gleichermaßen auf Gesundheit und auf Wohlbefinden beziehen. Unter salutogenetischer Perspektive ist diese weitgehende Gleichsetzung gerade bei seelischer Gesundheit auch legitim. Die günstige empirische Befundlage gilt allerdings für Erwachsene weitaus mehr als für Kinder. Belegt sind Effekte sowohl in somatischer als auch in psychischer und sozialer Hinsicht [1, 2, 20–22].

Dabei ist besonders die Natur in der unmittelbaren Wohnumgebung wirksam. Bei einer Auswertung von über 400.000 Krankenakten konnten Maas et al. [23] zeigen, dass Menschen in fußläufiger Nähe zu naturnahen Freiflächen ein höheres Wohlbefinden haben und auch objektiv gesünder sind: Sie haben weniger Kopfschmerzen, einen normaleren Blutdruck und leiden weniger an Atemwegserkrankungen und Allergien. Vor dem Hintergrund einer sehr großen Datenmenge konnten Krekel et al. [24] ebenfalls zeigen, dass mit der Nähe zu Grünanlagen die Lebenszufriedenheit steigt und Gesundheitsrisiken abnehmen. Dieser Zusammenhang von Erreichbarkeit von naturnahen Freiflächen und Wohlbefinden trifft auch auf Kinder zu [25]. Natürlich gibt es dabei noch viele offene Fragen. Zum Beispiel muss noch genauer geklärt werden, inwieweit die Erreichbarkeit von naturnahen Freiflächen auch mit dem sozioökonomischen Status zusammenhängt, der natürlich ebenfalls Gesundheit und Wohlbefinden bedingen dürfte. Außerdem haben die vielen hier zusammengetragenen Studien weder einen einheitlichen Naturbegriff noch einen expliziten Erfahrungsbegriff. Auch die Dauer und Häufigkeit der Naturkontakte ist unklar ebenso die Erfassung der Qualität der Naturumgebung.

Die meisten Studien zum Zusammenhang von Naturerfahrungen und Gesundheit beziehen sich auf Erwachsene. Natürlich ist anzunehmen, dass die Befunde bei Jugendlichen und Erwachsenen zumindest teilweise auf die Situation bei Kindern übertragbar sind, jedoch besteht hier noch eine ausgesprochene Forschungslücke. Im Folgenden werden ausgewählte Befunde im Hinblick auf Kinder zusammengestellt:

In einer Metaanalyse von Jimenez et al. [26] finden sich Zusammenhänge zwischen Naturerfahrung und verbesserter kognitiver Funktion, Gehirnaktivität, Blutdruck, psychischer Gesundheit, körperlicher Aktivität und Schlaf. Die Ergebnisse experimenteller Studien belegen die schützende Wirkung von Naturerlebnissen auf die psychische Gesundheit und auf kognitive Funktionen. Querschnittsuntersuchungen und Beobachtungsstudien belegen positive Zusammenhänge zwischen der Naturerfahrung und einem höheren Maß an körperlicher Aktivität sowie einem geringeren Risiko für Herz-Kreislauf-Erkrankungen. Und Längsschnittstudien belegen die langfristigen Auswirkungen von Naturerfahrungen auf Depressionen, Angstzustände, kognitive Funktionen und chronische Krankheiten.

In einer weiteren Metaanalyse [27] wurde deutlich, dass vermehrte Grünflächen in der Stadt bereits in der Schwangerschaft Wirkung zeigen. Es gibt hier einen Zusammenhang mit einem höheren Geburtsgewicht. Zudem ist gemäß dieser Metaanalyse eine höhere Grünflächenexposition in der (frühen) Kindheit entwicklungsfördernd; erreichbare Grünflächen sind mit einem höheren Maß an körperlicher Aktivität und einem geringeren Risiko für Fettleibigkeit und neurologische Entwicklungsprobleme wie Unaufmerksamkeit verbunden.

### Stressreduktion

Eine gut untersuchte Wirkung von Naturerfahrungen ist die Erholung von Stresssymptomen [28], und zwar auch schon bei Kindern [29]. Mygind et al. [30] konnten zeigen, dass regelmäßige Naturerfahrungen bei Schulkindern den Schulstress reduzieren und zudem die kognitive Entwicklung anregen [31]. Entsprechend der oben bereits genannten Stress Recovery Theory [12] befördern Naturerfahrungen positive Emotionen bzw. reduzieren negative Emotionen, wie etwa Stress. Das ist auch physiologisch nachweisbar (Herzfrequenz, Blutdruck, Cortisolausschüttung).

In einer Interventionsstudie [32] wurden die Veränderungen in stressbezogenen Gehirnregionen als Auswirkung eines einstündigen Spaziergangs in einer städtischen Umgebung (belebte Straße in Berlin) gegenüber einer natürlichen Umgebung (Wald) auch in neuropsychologischer Hinsicht untersucht. Die Hirnaktivierung wurde vor und nach einem Spaziergang mit Hilfe einer Aufgabe über ängstliche Gesichter und einer Aufgabe über sozialen Stress gemessen. Die Amygdala-Aktivierung nimmt nach einem Spaziergang in der Natur ab, während sie nach einem Spaziergang in einer städtischen Umgebung stabil bleibt. Ein Spaziergang in der Natur hat also einen Einfluss auf stressrelevante Hirnregionen und kann somit als Präventivmaßnahme gegen psychische Belastungen wirken.

### Antidepressive und stimmungsaufhellende Wirkung

Naturerfahrungen haben auch bereits bei Kindern eine antidepressive Wirkung. In einer groß angelegten dänischen Studie [33] konnte gezeigt werden, dass dies v. a. prognostisch günstig ist: Wenn Kinder (bis zu 10 Jahren) in nennenswertem Umfang Zugang zu Grünflächen haben und diese auch kontinuierlich nutzen, verringert dies die Prävalenz für die Entwicklung von psychiatrischen Störungen im Jugend- und Erwachsenenalter [33]. Der Untersuchung mit immerhin über 900.000 Personen zufolge gibt es diesbezügliche Effekte, außer für geistige Behinderung und schizoaffektive Störungen. Die Grünflächen haben einen maßgeblichen Einfluss: Durch Ausschlussverfahren konnte sichergestellt werden, dass die Effekte unabhängig von anderen Risikofaktoren wie Urbanisierung, sozioökonomische Faktoren, psychische Erkrankungen in der Familie und Alter der Eltern sind.

Überhaupt wirkt Natur als Stimmungsaufheller [34] indem positive Gefühle begünstigt werden und negative Gefühle abnehmen. Damit wird die seelische Gesundheit im Sinne von Mental Health und Well Being [35] positiv beeinflusst. Dabei ist der Zusammenhang von Naturerfahrung und Well Being bei 12- bis 13-Jährigen enger als bei 4‑ bis 5‑Jährigen [35].

### Prosoziales Verhalten

In einer 10-jährigen Längsschnittanalyse australischer Kinder konnte ein Zusammenhang zwischen Grünflächenqualität und prosozialem Verhalten gezeigt werden [36]. Jungen und jüngere Kinder profitierten tendenziell stärker davon. In einer italienischen Prä-Post-Studie im Kontext eines Umweltbildungsprojekts [37] wurde die Wirkung nicht nur auf das prosoziale Verhalten und empathische Fähigkeiten bestätigt, sondern auch auf das allgemeine Wohlbefinden.

Überhaupt werden soziale Fähigkeiten durch Naturerfahrungen entwickelt [38]. Kinder, die einen Waldkindergarten besuchen – also regelmäßig mit einer Gruppe draußen sind –, haben einen erfahreneren Umgang mit anderen Kindern [39]. Sie reagieren auch in schwierigen Situationen lösungsorientiert, produktiv, tolerant und kooperativ [40].

Das Eintauchen in eine naturnahe Umgebung führt zu einem Anstieg prosozialer Orientierungen und im Gegenzug zu einer Abnahme selbstbezogener Bestrebungen [41]. Vor dem Hintergrund der Selbstbestimmungstheorie der Motivation nehmen die Autoren der Studie an, dass das Eintauchen in die Natur das Autonomieerleben fördert. Das auf diese Weise gesicherte Autonomieerleben macht es dann auch eher möglich, von sich selber abzusehen.

### Aufmerksamkeit und ADHS

Aufenthalte in grünen Freiflächen (besonders im Wald) mildern die Symptome von chronischen Aufmerksamkeitsstörungen wie Aufmerksamkeitsschwächen oder Hyperaktivität [42]. Überhaupt sind Naturerfahrungen in der Lage, die Aufmerksamkeit zu fokussieren [43]. Zugleich verbessern sie die Konzentration. Es gibt danach einen Zusammenhang zwischen der Erreichbarkeit von grünen Freiflächen im Wohnumfeld und einer Verringerung der Prävalenz für die Aufmerksamkeitsdefizit‑/Hyperaktivitätsstörung (ADHS) um bis zu 33 %. Diese Ergebnisse werden erklärt vor dem Hintergrund der Attention Restoration Theory (s. oben).

Neuerdings gibt es hier allerdings Zweifel: In einer aktuellen randomisierten Studie mit 10-jährigen Kindern wurden die Effekte von Naturerfahrungen einerseits und Medikamenten andererseits auf ADHS verglichen [44]. Während die Medikation die Leistung verbesserte, gab es bei den Naturerfahrungen keinen Effekt. Die Unterschiede zu den vielfach beachteten früheren Studien von Faber Taylor und Kuo erklären die Autoren damit, dass die Effekte der Naturerfahrung auf die Symptome von ADHS in den älteren Studien von Eltern und Lehrkräften bewertet wurden, in der eigenen Studie jedoch durch objektvierbare Test- und Diagnoseverfahren kontrolliert erhoben.

### Kognitive Entwicklung

Es gibt Hinweise, dass überhaupt die kognitive Entwicklung durch regelmäßige Naturerfahrungen begünstigt wird [29–31, 45, 46]. Auch eine entsprechende Innenraumbegrünung (Biophilic Indoor Environment) ist für die kognitive Entwicklung bereits günstig [47].

### Selbstwert und Selbstregulation

Naturerfahrungen unterstützen das Selbstwertgefühl [29] ebenso die Selbstwirksamkeit [48]. In einer Studie mit „bildungsbenachteiligten“ Kindern und Jugendlichen [49] konnte in einem Prä-Post-Design gezeigt werden, dass sich durch regelmäßige Naturerfahrungen (wöchentlich über ein Jahr) die Selbstwirksamkeitserwartung gesteigert hat.

Interessant ist auch die Förderung der Selbstregulation durch Naturerfahrungen. Für Kindergartenkinder konnte in einem experimentellen Zugang gezeigt werden, dass die Entwicklung von selbstregulativen Fähigkeiten mit der Nutzung von Freiflächen zusammenhängt [50]. Ein täglicher Aufenthalt im Grünen erweist sich dabei als günstiger als ein wöchentlicher, v. a. bei Mädchen. Die Fähigkeit zur Selbstregulierung hängt den Autoren zufolge mit allgemeinem Wohlbefinden zusammen und auch mit schulischem Erfolg.

### Naturerfahrung und Bewegung

Grüne Freiflächen sind ein Anreiz zu physischer Aktivität [51, 52]; das gilt auch für Kinder [53] und physische Aktivität in Grünräumen wirkt sich günstiger aus als in anderen Räumen [54].

In einer schwedischen Studie [55] wurden Kindern der 2., 5. und 8. Klassen Sensoren-Armbänder appliziert, um ihr Bewegungsverhalten zu erfassen: An Schultagen verbrachten die Kinder über 2 Drittel in Gebäuden mit wenig Bewegungsmöglichkeiten, die Zweitklässler noch am meisten (zwischen 17 min und 129 min). Besonders interessant ist der Befund, dass alle Kinder sich nicht im Sportunterricht am häufigsten bewegten, sondern beim freien Spiel draußen.

Bei Bewegungserfahrungen in der Natur muss man sich den Anforderungen der jeweiligen Naturumgebung anpassen und es findet z. B. bei einem unebenen Walduntergrund ein ständiges Koordinationstraining statt [21]. Zu diesen psychomotorischen Effekten [21] gehört auch die Entwicklung der Gleichgewichtskontrolle.

Kinder, die immer wieder in naturnahen Freiflächen oder im Wald spielen, haben insgesamt eine bessere Motorik, sind fitter und auch weniger unfallgefährdet [56]. Es gibt einen Zusammenhang zwischen bebauter Umwelt, physischer Aktivität und Übergewicht bei Kindern [52, 56]. Natürlich beeinflussen auch andere Faktoren das Übergewicht, z. B. der sozioökonomische Status der Eltern.

## Beiläufige Wirkung von Naturerfahrungen und naturpädagogische Einflussmöglichkeiten

Der Zusammenhang von Naturerfahrung und Wohlbefinden wurde auch anhand von Kinderzeichnungen zu rekonstruieren versucht [57]. Dabei zeigt sich, dass für das Wohlbefinden haltende Sozialbeziehungen am wichtigsten sind. Natur kommt immerhin in über der Hälfte (56 von 91) der Zeichnungen vor, allerdings steht sie nur bei etwa jeder fünften im Vordergrund. Bei den meisten Bildern bildet die Natur „nur“ den Hintergrund, der überwiegend freundlich getönt ist. In den (wenigen) Zeichnungen, in denen Natur explizit zum Ausdruck kam, stand sie mit Entspannung, Kreativität und Spiel im Zusammenhang. Auch in anderen Studien wird Natur als Quelle von Wohlbefinden nur selten von Kindern explizit genannt [58]. Die offenbar v. a. im Hintergrund wirkende, implizite bzw. geradezu unbewusste Wirkung von Natur ist insofern interessant, als man daraus folgern kann, dass Naturerfahrungen sich weniger als gleichsam deterministisch wirksame, explizite, womöglich therapeutische Interventionen anbieten. Sie wirken vielmehr als beiläufiger Hintergrund in Form von grünen Freiräumen in fußläufiger Entfernung, der implizit Gesundheit und Wohlbefinden zu fördern in der Lage ist.

Diese Beiläufigkeit der Wirkung von Naturerfahrungen ist bei möglichen therapeutischen oder pädagogischen Interventionen zu bedenken. So ist bei erlebnis- oder naturpädagogischen Angeboten die Unverfügbarkeit von wahrhaftigen Erfahrungen [59] zu beachten. Es sind v. a. die Gefühle von Freiheit und Abenteuer, die bei Naturerfahrungen von Kindern gesucht und präferiert werden [1]. Eine allzu pädagogische Haltung würde die günstigen Wirkungen von Naturerfahrungen geradezu gefährden.

So hat sich in der bereits erwähnten Studie mit bildungsbenachteiligten Kindern und Jugendlichen [49] gezeigt, dass die folgenden pädagogischen Prinzipien sich günstig auf die Entwicklung von naturaffinen Einstellungsmustern und -bedürfnissen auswirken: Die Gewährung von Autonomie und Freizügigkeit (Partizipation), die Orientierung an Glücksmöglichkeiten in der Natur, ein damit verbundener Verzicht auf (umweltbezogene) Moralisierung und schließlich die Erschließung der symbolischen und sinnkonstituierenden Aspekte von Naturerfahrungen (s. Abschnitt „Natur als salutogener Faktor“).

Zur Ermöglichung bzw. Anbahnung von Naturerfahrungen gibt es in einigen Städten sogenannte Naturerfahrungsräume. Diese Naturerfahrungsräume sollen für die kindliche Entwicklung ein günstiges Gegengewicht angesichts der sogenannten veränderten Kindheit („Verhäuslichung“, „Verinselung“) darstellen. Nach Schemel ist ein städtischer Naturerfahrungsraum eine mindestens ein Hektar große „wilde“ Fläche in fußläufiger Nähe zur Wohnung, auf der frei ohne pädagogische Begleitung gespielt werden kann [60]. Diese „Naturerfahrungsräume“ [60, 61] sollen als Flächenkategorie im Bundesnaturschutzgesetz etabliert werden. In solchen Räumen (z. B. in Berlin [62]) ist das möglich, was in der Spielpädagogik „freies Spiel“ genannt wird, das übrigens in der Kinderrechtskonvention der Vereinten Nationen als Recht verbrieft ist.

Im Kontext der naturpädagogischen Einflussmöglichkeiten ist auch die Etablierung von sogenannten Draußenschulen [63] zu nennen. Während es in skandinavischen Ländern diesbezüglich schon eine lange Tradition gibt (Udeskole [64] oder Friluftsliv [65]), steht Deutschland am Anfang dieser Entwicklung. An der Laborschule Bielefeld läuft derzeit ein Modellprojekt, bei dem Kinder in nennenswertem Umfang freie Zeit draußen in der Natur verbringen [66]. Mit diesem Modellprojekt soll gezeigt werden, dass Naturerfahrungen im Kontext Schule nicht nur die Gesundheit, sondern auch Lern- und Bildungsprozesse befördern können.

Unabhängig von pädagogischen und bildungsbezogenen Ansätzen gibt es natürlich auch therapeutische Ansätze, die hier zumindest erwähnt werden sollen: So gibt es nicht nur Angebote mit Tieren [67], sondern auch entsprechende Versuche mit Pflanzen und Gärten [68]. „Therapeutische Landschaften“ [20] erzeugen Wohlbefinden und Gesundheit durch kontemplatives und aktives Naturerleben, und zwar v. a. angesichts der symbolischen und kulturellen Bedeutungen von Natur.

## Natur als salutogener Faktor

Naturerfahrungen haben etwas mit Wohlbefinden und Lebensqualität, mit einem guten Leben zu tun. Aus salutogenetischer Sicht [69, 70] kann man Natur und Landschaft als einen wirksamen Faktor betrachten, der uns in der Polarität zwischen Gesundheit und Krankheit in Richtung des Gesundheitspols orientiert. Durch diese salutogenetische Perspektive auf das Naturerleben gewinnen die symbolischen Bedeutungen von Natur ein besonderes Gewicht.

Die „Natur“ stellt gleichsam einen Symbolvorrat dar, der dem Menschen für Selbst- und Weltdeutungen zur Verfügung steht [71]. „Natur“ wird zu einem Symbol von Aspekten des eigenen Selbst oder – wie Caspar David Friedrich sagte – zur „Membran subjektiver Erfahrungen und Leiden“. Diese symbolische und kulturelle [72] Dimension unserer Naturbeziehungen ist für den Menschen als „animal symbolicum“ nicht unbedeutend, ist es doch gerade der symbolische Weltzugang, der es uns gestattet, unser Leben als ein sinnvolles zu interpretieren. Im Verhältnis zur äußeren Natur wird deshalb auch unser Verhältnis zu uns selbst sichtbar bzw. aktualisiert [7]. Dies wird für die heilsame und möglicherweise auch therapeutische Wirkung von Naturerfahrungen nicht unerheblich sein. Der Begriff der „therapeutischen Landschaften“ [20] zielt insofern auch nicht nur auf die physischen Attribute von Natur und Landschaft, sondern v. a. auf deren symbolische und kulturelle Bedeutung. Es gibt einen Zusammenhang von psychischer Gesundheit und dem Reichtum an symbolischen Bildern. Natursymbolisierungen (z. B. Wald, Wasser, Tiere) scheinen hier eine besondere Bedeutung zu haben.

Vor allem ambivalente Bedeutungen von Natursymbolen machen sie für eine psychodynamische Verwendung gut geeignet, weil widersprüchliche psychische Zustände einen symbolischen Anker finden können. Die Natur in ihren widersprüchlichen Eigenschaften ist für die nie von Ambivalenzen freie menschliche Seele ein Ort, an dem die inneren Ambivalenzen ihr bedrohliches Potenzial verlieren können. Indem die Natur sozusagen mit größter Selbstverständlichkeit Widersprüchliches, Ambivalentes, Spannungsreiches sowohl ist als auch – aufgrund der kulturellen Erzeugtheit von Naturbedeutungen [72] – symbolisch repräsentiert, kann sie zum symbolischen Hoffnungsträger dafür werden, dass sich innerseelische Widersprüche „aufheben“ lassen. Gerade angesichts besagter Ambivalenz ist jedoch zu bedenken, dass die positiven Konnotationen von Natur vor allem ein neuzeitliches Phänomen sind. So ist der Gedanke, dass der Mensch durch Naturerfahrungen auf eine geradezu wundersame Weise in seiner körperlichen, seelischen und sozialen Verfasstheit, in seinem Sinn- und Glücksbedürfnis sowie in seiner Gesundheit positiv berührt wird, eher eine romantische Idee. Dabei werden die bedrohlicheren Aspekte von Natur oft ausgeblendet. Die Nähe zur Natur setzt die Distanz zu ihr voraus, die erst durch Technik und Naturwissenschaft gewährleistet ist. Vor allem die gezähmte Natur wird als „schön“ wahrgenommen. So kann es durchaus als Privileg bezeichnet werden, dass der verstädterte Mensch der Moderne die Natur v. a. unter dem symbolisch-ästhetischen Aspekt gleichsam zweckfrei genießen kann.

Natur eignet sich offenbar dazu, innere Seelenzustände in äußeren Gegenständen zu symbolisieren. Das Erleben von äußerer heiler Natur kann heilsam auch für die innere Natur sein. So kann eine naturnahe und zugleich symbolisch bedeutungsvolle Umwelt dazu beitragen, das Kohärenzgefühl im Sinne der Salutogenese zu stärken. Eine solche naturnahe Umwelt hat zudem den Vorteil, dass sie relativ unerschöpflich ist und damit immer wieder zum Symbol eines geglückten, eines guten Lebens werden kann.
